# Toward Time-Resolved Analysis of RNA Metabolism in Archaea Using 4-Thiouracil

**DOI:** 10.3389/fmicb.2017.00286

**Published:** 2017-02-24

**Authors:** Robert Knüppel, Corinna Kuttenberger, Sébastien Ferreira-Cerca

**Affiliations:** Biochemistry III, Institute for Biochemistry, Genetics and Microbiology, University of RegensburgRegensburg, Germany

**Keywords:** archaea, RNA, 4-thiouracil, 4-thiouridine, 4TU, RNA-tagging, biotin

## Abstract

Archaea are widespread organisms colonizing almost every habitat on Earth. However, the molecular biology of archaea still remains relatively uncharacterized. RNA metabolism is a central cellular process, which has been extensively analyzed in both bacteria and eukarya. In contrast, analysis of RNA metabolism dynamic in archaea has been limited to date. To facilitate analysis of the RNA metabolism dynamic at a system-wide scale in archaea, we have established non-radioactive pulse labeling of RNA, using the nucleotide analog 4-thiouracil (4TU) in two commonly used model archaea: the halophile Euryarchaeota *Haloferax volcanii*, and the thermo-acidophile Crenarchaeota *Sulfolobus acidocaldarius*. In this work, we show that 4TU pulse labeling can be efficiently performed in these two organisms in a dose- and time-dependent manner. In addition, our results suggest that uracil prototrophy had no critical impact on the overall 4TU incorporation in RNA molecules. Accordingly, our work suggests that 4TU incorporation can be widely performed in archaea, thereby expanding the molecular toolkit to analyze archaeal gene expression network dynamic in unprecedented detail.

## Introduction

Determining the function of every single gene remains a challenging task for modern biology. The rise of –omics analyses has provided a great deal of information, which still needs to be integrated into meaningful cellular and molecular functions. In the post-genomic era, several functional analysis strategies can be applied to unravel the information stored at the DNA level. However, comprehensive functional analysis of individual genes contained in an individual genome still remains a daunting task (e.g., Bork et al., 1998; Niehrs and Pollet, 1999; Wu et al., 2002; Schlitt et al., 2003; Fraser and Marcotte, 2004). Moreover, complex phenotypic traits rely essentially on the establishment of highly cooperative and dynamic gene networks that can integrate the sum of functions of several individual gene products at a given time (e.g., Barabasi and Oltvai, 2004; Fraser and Marcotte, 2004). Whereas, classical functional genomic analysis can help to determine the function of a gene, such analysis often relies on prior knowledge and/or hypothetical projection (for example, predictions based on gene sequence similarities). Consequently, and despite full genome sequencing of numerous organisms, the function of many open reading frames remains poorly characterized.

System-wide profiling of gene expression and gene expression networks can aid in attributing functions to a gene, or groups of genes (e.g., Gardner et al., 2003; Ihmels et al., 2004; Blais and Dynlacht, 2005). Recently, the determination of the relative abundance of RNA by high-throughput technologies has revealed an unprecedented depth of information (Wang et al., 2009). Together, these key technologies provide fundamental information to predict and rationalize the molecular mechanisms that account for the formation of specific cellular phenotypes. However, the inherent lack of dynamic analysis in such steady-state assays does not grant full-depth understanding of the molecular basis enabling the formation of complex phenotypes (e.g., Friedel and Dolken, 2009).

To overcome such limitations, dynamic gene-expression network profiling has been applied to better characterize the gene expression networks accounting for the dynamic expression of complex traits in relation to environmental cues (e.g., Dölken et al., 2008; Zeiner et al., 2008; Friedel and Dolken, 2009; Miller et al., 2009; Tallafuss et al., 2014).

RNA-tagging approaches with the use of nucleotide/ nucleoside analogs, and in combination with next generation sequencing, is one of the recent technological developments that allows powerful analysis of RNA synthesis and degradation rates in a time- and condition-dependent manner. Nucleotide analogs, such as 4-thiouracil- (4TU) or 4-thiouridine- (4SU), -based RNA-tagging has been successfully applied in various cell types and tissues in combination with high-throughput methodologies (e.g., Favre et al., 1986; Dölken et al., 2008; Zeiner et al., 2008; Friedel and Dolken, 2009; Miller et al., 2009; Tallafuss et al., 2014). Together, these studies have provided essential information for further understanding of RNA metabolism and its dynamics (e.g., Swiatkowska et al., 2012; Burger et al., 2013; Barrass et al., 2015; Duffy et al., 2015; Hulscher et al., 2016). Moreover, the recent improvement of RNA-tagging chemistry enables a better enrichment of the tagged-RNA population providing additional perspectives to more precisely characterize gene-expression networks (Duffy et al., 2015). Furthermore, the promising usage of nucleotide analogs in combination with photo-crosslinking approaches allows the systematic analysis of RNA binding protein (RBP)-RNA interactions and to decipher the RBP repertoire (Hafner et al., 2010; Ascano et al., 2012; Castello et al., 2012). Whereas, nucleotide analog-based RNA-tagging has emerged as a key technology to systematically decipher fundamental aspects of RNA biology, surprisingly 4TU/4SU labeling, to our knowledge, has not been established in any archaea.

Since we are interested in deciphering key molecular principles of RNA metabolism in archaea, we sought to fill this methodological gap by first establishing and applying 4TU labeling in archaea. As a proof of principle we have employed 4TU labeling in two genetically-tractable representative archaeal organisms—*Haloferax volcanii* and *Sulfolobus acidocaldarius*—thereby expanding the archaeal molecular biology toolkit.

## Results

### *In vivo* Incorporation of 4TU in Exemplary Archaea

We initially speculated that efficient incorporation of 4TU into RNA molecules would largely depend on the following parameters: (i) efficiency of intra-cellular import of exogenous nucleobase, (ii) presence of enzyme(s) allowing conversion of the nucleobase into nucleotide tri-phosphate, (iii) and the existence of defined cultivation procedures where the nucleobase can be exogenously added.

On the basis of these criteria, we have selected widely used, easily cultivable model organisms representative of two major archaeal phyla: the Euryarchaeota *Haloferax volcanii*, and the Crenarchaeota *Sulfolobus acidocaldarius*. Both organisms can be cultivated aerobically in distinct media where the source of uracil can be exogenously added and controlled (Atomi et al., 2012). Moreover, in both organisms the pyrimidine biosynthesis pathway has been successfully mutated, whereby the respective *pyrE* genes encoding orotate phosphoribosyltransferase have been knocked out (Allers et al., 2004; Wagner et al., 2012). In this context, cells exclusively rely on an exogenous source of uracil to maintain growth.

**FIGURE 1:**
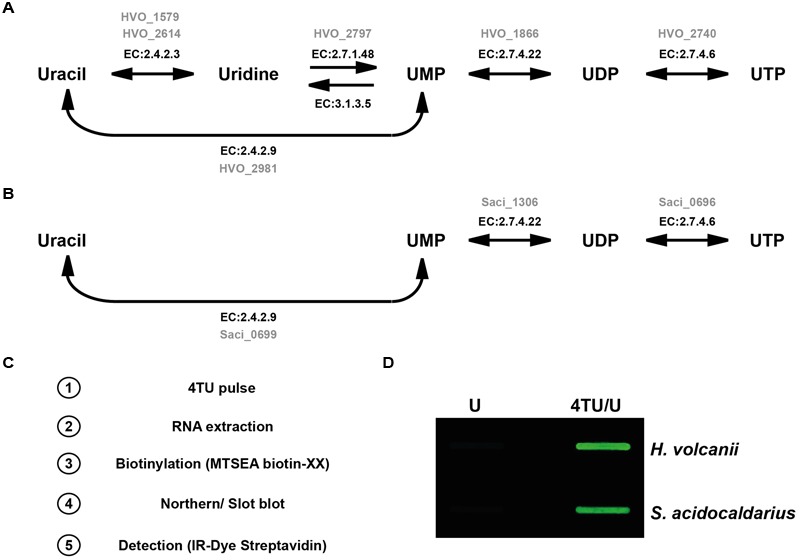
***In vivo* incorporation of 4TU in model archaea. (A)** Synthesis pathway of UTP from uracil in *Haloferax volcanii* is depicted according to KEGG pyrimidine synthesis pathway (KEGG entry: hvo00240) (Kanehisa et al., 2016). Enzyme classification number (E.C) and their corresponding open reading frame in *H. volcanii* (in gray) encoding the enzyme activity are indicated. **(B)** Synthesis pathway of UTP from uracil in *Sulfolobus acidocaldarius* is depicted according to KEGG pyrimidine synthesis pathway (KEGG entry: sai00240) (Kanehisa et al., 2016). Enzyme classification number (E.C) and their corresponding open reading frame in *S. acidocaldarius* (in gray) encoding the enzyme activity are indicated. **(C)** 4-thiouracil (4TU) labeling and detection work flow. **(D)** Analysis of 4TU incorporation in *H. volcanii* and *S. acidocaldarius*. *H. volcanii* (H26) and *S. acidocaldarius* (MW001) cells were grown for several generations either in medium containing a mixture of 4-thiouracil and uracil (4TU/U - 3:1) or in medium solely containing uracil (U) as described in the Section “Materials and Methods”. Biotinylated uracil was detected by infra-red fluorescence.

Finally, conversion of the thiouracil into nucleotide-triphosphate (thio-UTP) is critically dependent on the uracil phophosribosyltransferase (EC: 2.4.2.9). In addition, in contrast to most multicellular eukaryotic organisms, with the exception of *Toxoplasma gondii*, only bacteria and unicellular eukaryotes have been shown to be able to convert thiouracil into thio-UMP, using their endogenous uracil phophosribosyltransferase (Cleary et al., 2005). As summarized in **Figure 1**, both organisms contain the annotated genes allowing the conversion of (thio-) uracil into (thio-) UTP (Kanehisa et al., 2016).

Since both organisms presented all the critical characteristics mentioned above to putatively allow efficient 4TU incorporation into RNA molecules, we next cultivated them in presence of 4TU over several growth generations to ensure sufficient intra-cellular accumulation of the nucleobase analog. Initially, we arbitrarily grew the cells in growth medium containing a 1:3 mixture of uracil and 4TU (75% 4TU) respectively, (see Section “Materials and Methods”). Moreover, during the course of the experiments, the growth of the cells cultivated with or without 4TU was apparently indistinguishable at this working concentration (see below for detailed toxicity analysis). Nucleic-acids (total RNA) from cells grown in presence and absence of 4TU were extracted by hot-phenol/chloroform extraction (see Section “Materials and Methods”). The 4TU-labeled RNAs were then biotinylated (with HPDP-biotin or MTSEA-XX-biotin). Biotinylated-RNAs were immobilized on a nylon membrane prior to detection with the help of streptavidin conjugated to an infra-red dye and visualized on a LI-COR Odyssey imaging device (see **Figure 1C** for work flow and Section “Materials and Methods” for the detailed protocol).

As shown in **Figure 1D**, 4TU enrichment was observed in both organisms. Moreover, in agreement with a recent report (Duffy et al., 2015), the detection sensitivity was strongly enhanced by the use of MTSEA-biotin-XX (**Supplementary Figure S1**).

### Incorporation of 4TU into RNA Polymers

**FIGURE 2:**
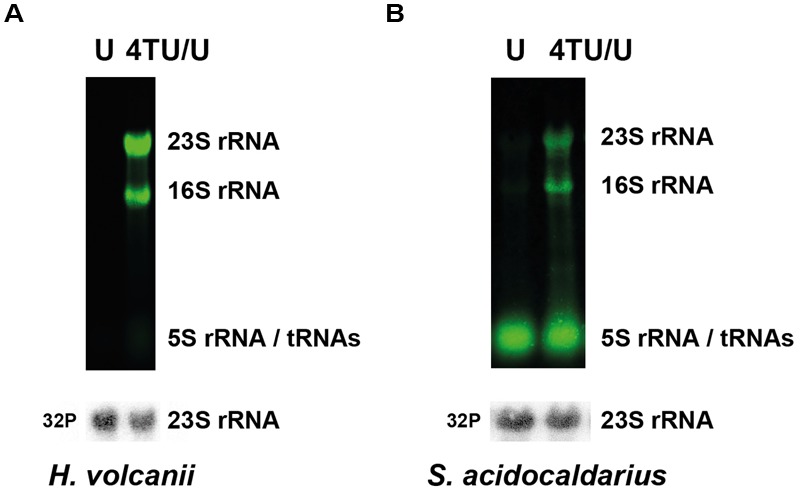
***In vivo* incorporation of 4TU in RNA polymers.** Steady-state incorporation of 4TU in abundant RNA molecules (rRNAs and tRNAs) in **(A)** *H. volcanii* and **(B)** *S. acidocaldarius* is depicted. Cells were grown over-night in culture medium either solely containing uracil (U) or containing a defined mixture of 4-thiouracil and uracil (4TU/U – 3:1). Abundant 4TU-containing RNA species were detected by infra-red fluorescence (upper panel – green). Bulk steady-state 23S rRNA (used as loading control) was detected using ^32^P radiolabeled antisense oligo probes (lower panel – gray) as described in the Section “Materials and Methods”.

Accumulation of intracellular 4TU is *per se* not an indication for proper incorporation of the nucleotide analog into functional RNA polymers. Therefore, to demonstrate incorporation of 4TU into RNA polymers, we monitored the presence of 4TU in stable abundant RNA (i.e., rRNA and tRNA). To this end, biotinylated total RNAs were separated by denaturing-agarose gel electrophoresis. As shown in **Figure 2**, the major stable RNAs are readily labeled by 4TU. Collectively, these experiments demonstrate the feasibility to perform 4TU incorporation into RNA molecules in archaea.

### 4TU is Toxic at Higher Concentrations

**FIGURE 3:**
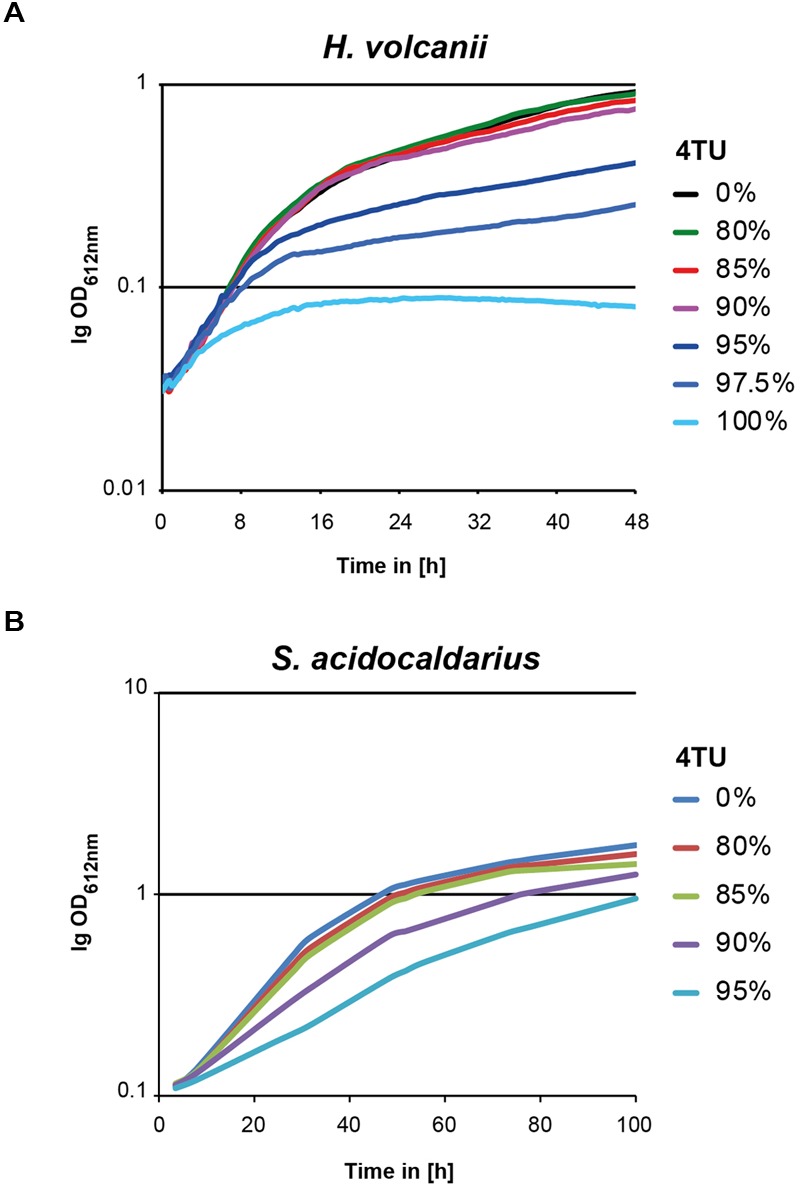
***In vivo* toxicity of 4TU in model archaea.** Growth analysis of **(A)** *H. volcanii* and **(B)** *S. acidocaldarius* grown in presence of different ratios of 4TU to uracil are depicted. Logarithmically growing cells were inoculated in culture medium containing the indicated amounts (in %) of 4TU/uracil. Growth was further monitored by measuring optical density at regular time intervals over the depicted time. Representative results are provided.

A previous report in mammalian cells demonstrated the potential toxic effect of 4TU incorporation on cellular growth (Burger et al., 2013). To evaluate this possibility, we grew *H. volcanii* and *S. acidocaldarius* cells in conditions where the final concentration of uracil-(analog) source (uracil/4TU) was constant, whereas the ratio of uracil/4TU was varied. *H. volcanii* growth curves are depicted in **Figure 3A**, and were generated in 96-well plate format [as described in Jantzer et al. (2011) and Section “Materials and Methods”]. In contrast, *S. acidocaldarius* growth analyses were performed manually and are depicted in **Figure 3B**. These growth analyses demonstrate little to no effect on growth behavior to up to 80% of 4TU as total source of uracil for both organisms. However, higher concentrations of 4TU significantly inhibited growth of both *H. volcanii* and *S. acidocaldarius*. This suggests that higher concentrations of 4TU are toxic. Therefore, for our subsequent analysis, we used concentrations of 4TU equal to or below 75% of the total uracil source.

### Time-Dependent 4TU Labeling of Major Cellular RNA

**FIGURE 4:**
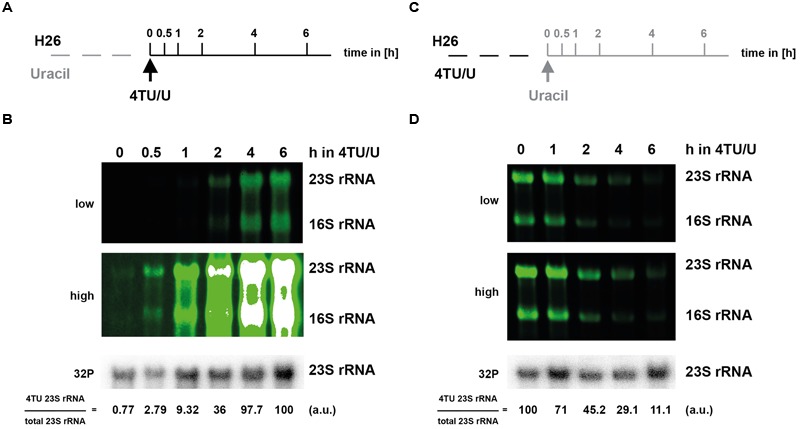
**Time-dependent incorporation of 4TU in *H. volcanii* RNA molecules.** 4TU pulse and pulse-chase experiments in *H. volcanii* are depicted in panel **(A–D)**, respectively. **(A)** 4TU pulse-labeling experimental work flow. Logarithmically growing cells were transferred in pre-warmed culture medium containing 75% of 4TU as uracil source (*t* = 0). Aliquots were collected at the indicated time points after addition of 4TU. **(B)** Time-dependent incorporation of 4TU in 16S and 23S rRNA. Bio-tagged-RNAs and bulk 23S rRNA (upper panels – in green and lowest panel – in gray, respectively) were visualized and quantified as described in the Section “Materials and Methods”. Two different acquisition sensitivities of the fluorescent signals are shown (low and high). Note that the white signals indicate fluorescence signal acquisition saturation. **(C)** 4TU pulse-chase labeling experimental work flow. *H. volcanii* cells were kept logarithmically growing in culture medium containing 75% of 4TU as total uracil source for 18 h to ensure labeling of most of the stable rRNA molecules (*t* = 0). Cells were then transferred to medium lacking 4TU and harvested at the indicated time points. **(D)** Time-dependent decrease of 4TU incorporation in 16S and 23S rRNA. RNA was analyzed as described in **(B)**. (a.u.) arbitrary units.

**FIGURE 5:**
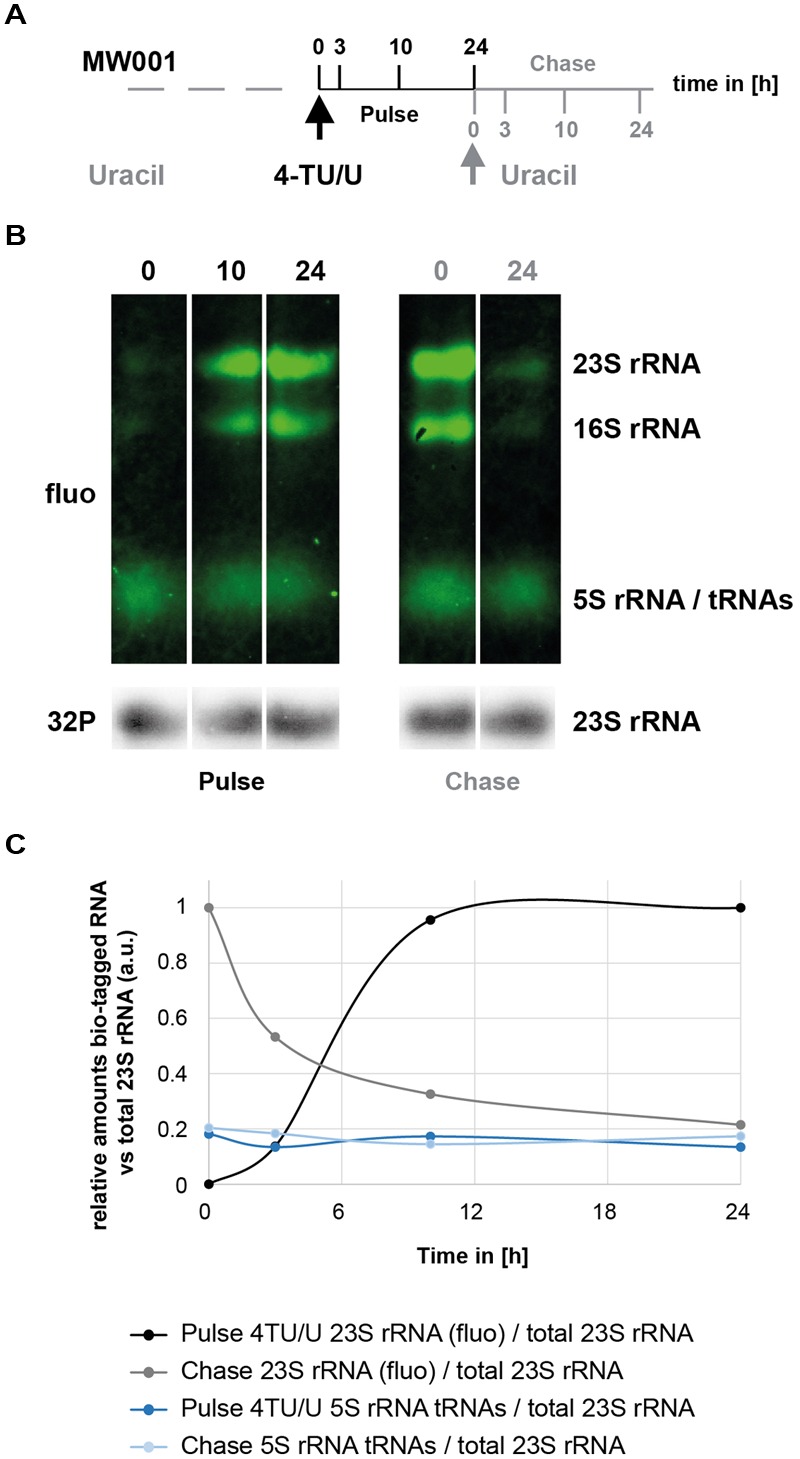
**Time-dependent incorporation of 4TU in *S. acidocaldarius* RNA molecules. (A)** 4TU pulse- and chase-labeling experimental work flow. Logarithmically growing cells were first labeled with 4TU for 24 h (Pulse) and chased for another 24 h (Chase). Aliquots were collected at the indicated time points. **(B)** Time-dependent incorporation of 4TU in stabile RNA. Bio-tagged RNAs (upper panels – in green) and total 23S rRNA (lower panel – in gray) were visualized and quantified as described in the Section “Materials and Methods”. Representative time-points are provided. **(C)** Relative quantification of the major bio-tagged RNA in *S. acidocaldarius*. Relative amounts of bio-tagged RNA species after 4TU pulse-labeling and chase were quantified overtime in relation to total 23S rRNA (quantified from data depicted in **(B)** and data not shown).

One critical advantage of 4TU labeling is the possibility to perform pulse and pulse-chase labeling, thereby allowing time-dependent analysis of RNA synthesis/decay at a given condition. To evaluate this opportunity, we performed pulse and pulse-chase labeling in *H. volcanii* and *S. acidocaldarius* (**Figures 4** and **5**, respectively). Interestingly, in *H. volcanii*, 4TU incorporation in the large rRNA (16S and 23S) was detectable at a relatively short-time point (30 min; 10–15% of a typical cell cycle in these growth conditions), which strongly accumulated over time (**Figure 4**). Likewise, pulse and pulse-chase experiments were performed in *S. acidocaldarius* (**Figure 5**). However, we noticed the presence of a stable population of shorter RNAs, presumably containing thio-modified nucleoside residues in this organism (see **Figure 5** and Section “Discussion”). Finally, pulse-chase experiments demonstrated a relative decrease of the labeled rRNA over time (**Figures 4** and **5**). Altogether, these results highlight the feasibility of performing 4TU-based time-dependent analysis of RNA metabolism in archaea.

### Relative 4TU-Labeling Efficiency

**FIGURE 6:**
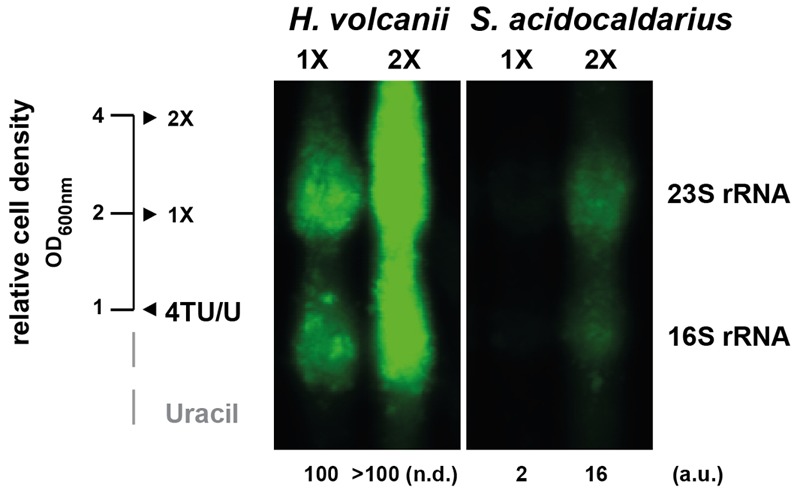
**Relative incorporation efficiency of 4TU in *H. volcanii* and *S. acidocaldarius*.** Logarithmically growing cells (H26 and MW001) were pulse-labeled over two generations as monitored to the relative increase of cell density. After each cell population doubling (1X and 2X) aliquots were taken and analyzed as described in the Section “Materials and Methods”. For comparison, equal amounts of total RNA were loaded. Relative quantification of 4TU-labeled 23S rRNA are provided. (a.u.) arbitrary units, (n.d.) non-determined.

In our labeling experiments, we noticed that the apparent labeling efficiency in *S. acidocaldarius* was reduced as compared to that in *H. volcanii*. However, since the cells had different doubling times (∼3 h for *H. volcanii* and ∼9 h for *S. acidocaldarius)* in these growth conditions, it was difficult to perform an accurate comparison of the 4TU labeling efficiencies in the conditions described above. Therefore, to perform a fair comparison of the relative 4TU labeling efficiencies in these organisms we sought to minimize the growth rate-dependent effects on the overall incorporation efficiency. To this end, we labeled cells during one and two full generation times. In addition, equal amounts of total RNA were analyzed. As shown in **Figure 6**, the total fraction of labeled rRNA present in *S. acidocaldarius* is significantly reduced compared to that in *H. volcanii*. This result indicates, that despite successful labeling of both organisms, the overall labeling efficiencies may vary between different archaea.

### Optimal 4TU Concentrations

**FIGURE 7:**
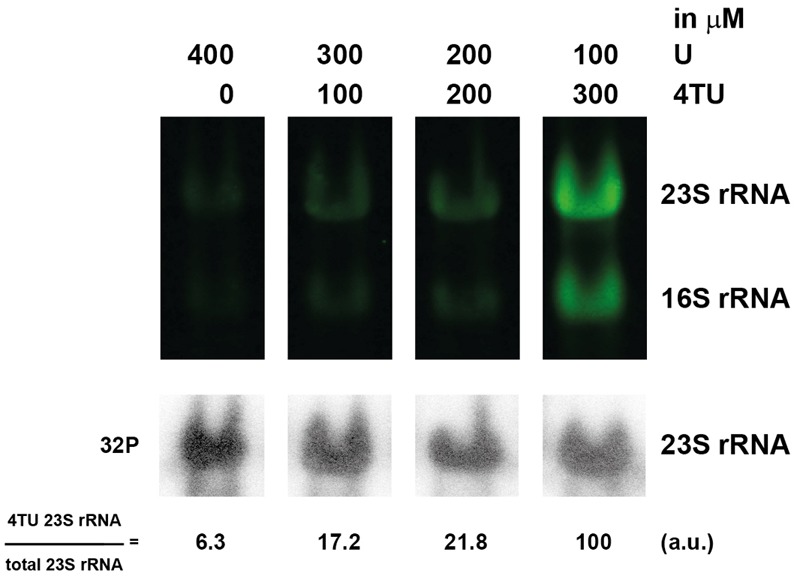
**Optimal concentration of 4TU in *H. volcanii*.** Logarithmically growing cells were grown in culture medium containing the indicated amounts of 4TU and uracil for 2 h. RNAs were analyzed and quantified, as described in the Section “Materials and Methods”.

Next, we sought to further optimize the 4TU labeling conditions. For this purpose, we analyzed the influence of various 4TU concentrations on the detection of synthesized tagged-RNA in *H. volcanii*. To this end, time-dependent pulse labeling with various amounts of 4TU (25, 50, and 75% of total uracil source) was performed (**Figure 7**). This analysis shows that within the non-toxic range of 4TU concentrations tested, the use of 75% of 4TU as total uracil allows robust labeling of RNA.

### Influence of Pyrimidine *de Novo* Synthesis on Overall 4TU Incorporation Efficiency

**FIGURE 8:**
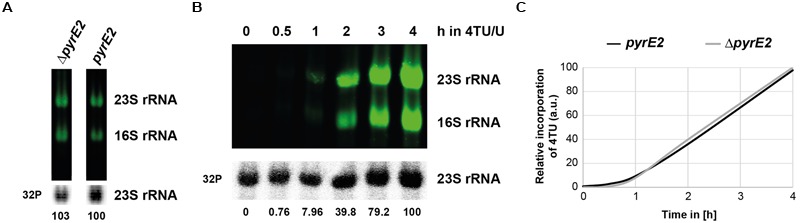
**Influence of pyrimidine *de novo* synthesis on overall 4TU incorporation. (A)** 4TU incorporation in uracil auxotroph and prototroph strains. Uracil auxotroph and prototroph (*pyrE2::HVO_0569*) strains growing in Hv-Ca^+^ medium supplemented or lacking uracil, respectively, were transferred for 8 h in pre-warmed Hv-Ca^+^ medium containing 75% 4TU as uracil source. RNA were analyzed and quantified as described in the Section “Materials and Methods”. Results of relative quantification (4TU-labeled 23S rRNA/total 23S rRNA) are provided **(B)** Time-dependent incorporation of 4TU in 16S and 23S rRNA in uracil prototroph strain. Same as in **(A)**, except that cells were grown in pre-warmed Hv-Ca^+^ medium containing 75% 4TU as uracil source and harvested at the indicated time-points. **(C)** Relative 4TU incorporation efficiency in auxotroph and prototroph cells. Results of relative quantification of 4TU incorporation in 23S rRNA from uracil auxotroph (Δ*pyrE2*) (taken from **Figure 4B**) and from uracil prototroph (*pyrE2::HVO_0569*) (**Figure 8B**) strains are depicted.

Cells used in this study have been engineered by genomic deletion of the *pyrE* gene essential for *de novo* synthesis of pyrimidine. However, for many other archaea uracil auxotroph strains are not always available. The presence of a *de novo* synthesis pathway could lead to a lower usage efficiency of the extra-cellular uracil/4TU. Therefore, we evaluated incorporation of 4TU in a situation where the *pyrE* gene, and thus the pyrimidine *de novo* synthesis pathway, is restored (see **Figure 8**). We derived an H26 strain where the *pyrE2* gene has been genomically integrated at a heterologous locus (see Section “Materials and Methods” and **Supplementary Figure S2**). The resulting uracil prototroph cells were grown in medium lacking uracil prior to addition of 4TU/U (3:1) and aliquots were taken at the indicated time points. As shown in **Figure 8**, labeling of uracil prototroph strain efficiency was similar to that obtained in the isogenic uracil auxotroph strain. Therefore, we conclude that 4TU labeling can be potentially widely achieved in most archaea that can be grown under defined laboratory conditions.

### Analysis of the Relative Transcriptional Activity of a Regulated mRNA, Using 4TU

**FIGURE 9:**
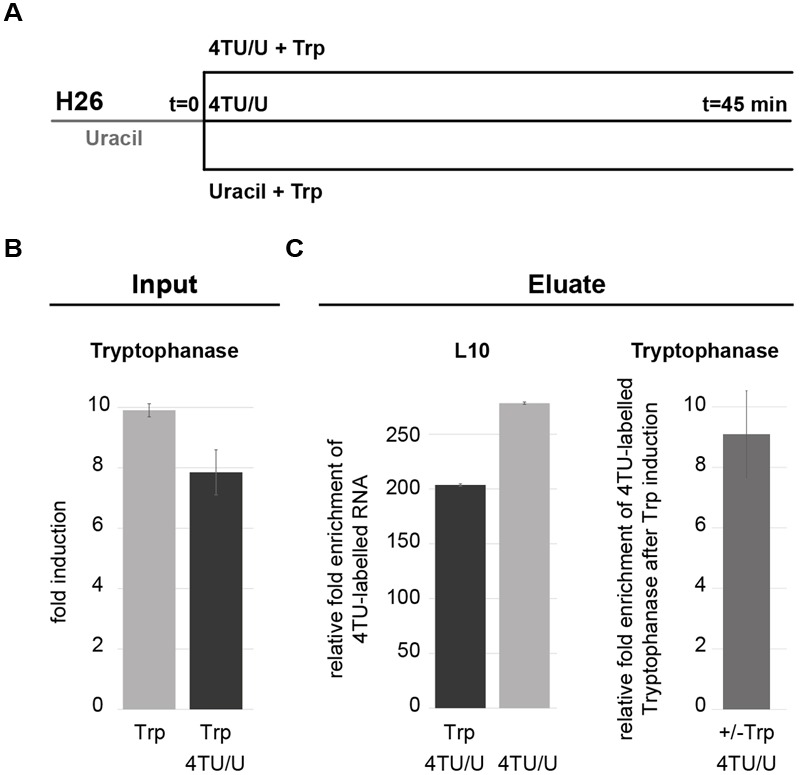
**Analysis of the transcriptionally regulated tryptophanase mRNA with 4TU. (A)** Experimental work flow. Cells were grown in Hv-Ca+ medium containing uracil and lacking L-Tryptophan. At the indicated time point (*t* = 0) cells were split and incubated for 45 min in presence of 4TU with or without 1 mM L-Tryptophan (Trp) or Hv-Ca+ medium containing uracil and L-Tryptophan. Bio-tagged RNA were purified by affinity purification and analyzed by qRT-PCR. **(B)** Induction of tryptophanase transcription by L-Tryptophan. Fold induction of tryptophanase in cells grown with Trp and with or without 4TU is depicted. Expression levels were normalized to the expression level obtained from cells grown without Trp. **(C)** Analysis of affinity purified 4TU-labeled mRNA. Left-panel: relative purification efficiency was obtained by comparing the enrichment of the housekeeping ribosomal protein L10 mRNA from cells labeled with 4TU with or without Trp and normalized to L10 mRNA from cells grown in absence of 4TU (background control). Right-panel: relative fold enrichment of 4TU-labeled tryptophanase after Trp induction were obtained by comparing the relative amount of purified 4TU-labeled tryptophanase after Trp induction and the relative amount of purified 4TU-labeled without Trp. Values were normalized to the amount of purified 4TU-labeled L10 mRNA obtained with and without Trp.

4TU labeling has been widely applied for the analysis of short-lived and/or less abundant transcripts such as mRNAs. In this case, the 4TU-labeled RNA needs to be enriched by affinity purification before analysis. To ensure that 4TU pulse labeling can be applied for the analysis of archaeal mRNA we analyzed the transcriptional response of the regulated mRNA encoding tryptophanase (Large et al., 2007). This previous study has shown that upon addition of L-Tryptophan transcription of the tryptophanase gene is up-regulated (Large et al., 2007). Using this regulated mRNA as a model system, we performed 4TU pulse labeling over the course of addition of L-Tryptophan (**Figure 9A**). As shown in **Figure 9** the relative amount of the tryptophanase mRNA in the 4TU-enriched RNA fraction (**Figure 9C** – right panel) mirrors the one observed in the total RNA fraction (Input) (**Figure 9B**). Together, these results show that the transcriptional state of 4TU-labeled mRNA can also be analyzed in archaea.

## Discussion

In this work, we provide proof of principle for the application of 4TU-based RNA-tagging in archaea. Given the known advantages of the methodology to analyze RNA dynamics and, RNA-protein interactions (e.g., Hafner et al., 2010; Ascano et al., 2012; Castello et al., 2012; Swiatkowska et al., 2012; Tallafuss et al., 2014; Duffy et al., 2015; Hulscher et al., 2016), we are confident that our proof of principle analysis broadens the archaeal molecular biology toolkit and will stimulate deeper analysis of RNA dynamics and gene expression networks in archaea.

We provide evidence that time-dependent 4TU based RNA-tagging can be widely applied in different archaeal organisms independently of their uracil auxotrophy or prototrophy. However, one of the most critical parameters allowing efficient 4TU labeling appears to be the ability to grow the corresponding archaeal organisms in controlled drop-out medium lacking uracil. Moreover, the intrinsic 4TU uptake and/ or stability of the nucleotide analog in a particular growth conditions might influence the overall labeling efficiencies.

Whereas high amounts of 4TU provided in the culture medium are apparently well-tolerated in *H. volcanii* and *S. acidocaldarius*, it should be noted that, owing to the inherent toxicity of 4TU, the ratio of 4TU to uracil chosen is critical. A previous study, has suggested that higher amounts of 4-thiouridine provided to human cell culture models have toxic effects, whereby early steps of ribosome synthesis are affected (Burger et al., 2013). Moreover, deleterious cross-talk effects of 4TU on naturally occurring uridine modifications (e.g., thiouridine derivatives, pseudouridine) have not been properly evaluated to date. Therefore, toxicity behavior for the respective organisms should be first determined empirically.

Another possible limitation of 4TU-based RNA tagging is the presence of free sulfhydryl groups, mostly found in the form of thiouridine modifications in tRNAs (2- and 4-thiouridine and their derivatives) (Tomikawa et al., 2013; Machnicka et al., 2014). In this case, the presence of endogenous sulfur-modified nucleosides will react with the modified biotin moiety and can potentially interfere with downstream experiments. Therefore, background reactivity should be carefully evaluated first. However, the complement and repartition of tRNA modifications in archaea is not yet fully characterized. Interestingly, whereas 4-thiouridine modification was not detected in *H. volcanii* tRNAs (Phillips and de Crécy-Lagard, 2011), 2-thiouridine modifications occurring at the wobble position of tRNA for lysine (tRNA^Lys^_UUU_), glutamate (tRNA^Glu^_UUC_), and glutamine (tRNA^Gln^_UUG_) have been only formally established for tRNA^Glu^_UUC_ and tRNA^Lys^_UUU_ in *H. volcanii* (Rogers et al., 1995; Miranda et al., 2011; Chavarria et al., 2014; Shigi, 2014). In agreement, we have not observed significant labeling of tRNA in absence of 4TU in *H. volcanii* (see **Figure 2A**). In contrast, a substantial population of sulfur-modified (t)RNA was detected in the absence of 4TU labeling in *S. acidocaldarius* (**Figures 2B** and **5**). As described above, 2-thiouridine modification is known to be restricted to a small population of tRNAs, whereas most bacterial tRNAs contain 4-thiouridine (Rogers et al., 1995). Therefore, the relative high amounts of thiolated tRNA could reflect a high steady-state level of endogenous 4-thiouridine-modified tRNA in *S. acidocaldarius*. The biosynthesis of 4-thiouridine is still not completely resolved in archaea. Recently, a conserved C*XX*C motif located in the PP-loop domain of *Methanococcus maripaludis* ThiI (MMP1354) has been shown to be essential for both *in vitro* and *in vivo* 4-thiouridine formation (Liu et al., 2012). Surprisingly, Halobacteriales and Sulfolobales are missing this critical CXXC motif (Liu et al., 2012; Tomikawa et al., 2013), suggesting that this group of organisms either lack 4-thiouridine modification (as demonstrated in *H. volcanii*) or use different or unknown mechanisms for the formation of 4-thiouridine. Another possibility is that *S. acidocaldarius* contains other type(s) of sulfur-containing nucleosides (e.g., 2-thioribothymidine) that contribute to its high-temperature adaptation (Edmonds et al., 1991; Shigi, 2014). Future studies will be needed to establish the nature and targets of the thio-modification observed in *S. acidocaldarius*.

Finally, biorthogonal amino acid pulse-labeling in combination with fluorescence *in situ* hybridization was developed to characterize cell-specific translational activity within complex environmental samples (Hatzenpichler et al., 2016, 2014). Whereas, we show that 4TU based RNA-tagging seems to be amenable for most organisms grown under laboratory conditions, we speculate that 4TU labeling could be also used beyond its classical “laboratory usage” to characterize microbial consortia. In this context, using advantages of 4TU-tagged RNA sequencing analysis would provide complementary information about the organism-specific rate of rRNA synthesis, thereby shedding light on both the composition and the relative growth dynamic of the microbial consortia.

## Conclusion

We described, proof of principle and workflow allowing the analysis of the RNA dynamic and gene network regulation in a time-dependent manner in archaea. The versatility and robustness of 4TU-based RNA-tagging can now be implemented in archaea and will further allow to elucidate the archaeal RNA metabolism.

## Materials and Methods

### Strains, Plasmids, and Growth Conditions

*Haloferax volcanii* strains (H26 Δ*pyrE2* – (Allers et al., 2004) and *pyrE2::HVO_0569* pop-in, this work) were grown in enhanced casamino acids media [Hv-Ca^+^: Hv-casamino acids medium supplemented with carbon source (Allers et al., 2004)] at 42°C under vigorous agitation. *S. acidocaldarius* [MW001 Δ*pyrE* – (Wagner et al., 2012)] cells were grown in standard Brock medium (Brock et al., 1972; Wagner et al., 2012) at 65°C under vigorous agitation.

Molecular cloning and amplification of plasmids were performed according to standard molecular biology methods.

### Construction of Pyrimidine Prototroph Strain

Uracil prototroph strain was obtained by genomic integration of the *pyrE2* gene at the HVO_0569 locus^1^ using the pop-in strategy (Allers et al., 2004) (**Supplementary Figure S2**). For homologous recombination, 500 bp of the upstream and downstream regions spanning the HVO_0569 open reading frame were amplified by PCR using the following primers: us: oHv_025: 5′-GCATCGAGGGTACCTCGTCGTGCGAATCGCGGACG-3′ and oHv_026: 5′-AGCGGAGGGGTCTCGACGGCGGATCCGCACGTCCGTCCGTCGGCGA-3′; ds: oHv_027 : 5′-TCGCCGACGGACGGACGTGCGGATCCGCCGTCGAGACCCCTCCGCT-3′ / oHv_028: 5′-CTAGCCACTCTAGAGACCTCGCGCTCGACGGCGTGGC-3′ (underlined nucleotides indicate the restriction enzyme sites used for cloning), and cloned into the integrative vector (pTA131) containing the *pyrE2* gene as selection marker, as previously described (Allers et al., 2004). The resulting integrative plasmid was transformed in H26 cells as previously described (Allers et al., 2004), and positive transformants were selected on Hv-Ca^+^ plates lacking uracil.

### 4-TU Toxicity Analysis

Semi-automated growth analysis was performed as previously described (Jantzer et al., 2011). In brief, exponentially growing cells (Hv-Ca^+^ containing uracil) were diluted in Hv-Ca^+^ media containing various relative amounts of 4TU/uracil (final concentration 400μM) and aliquoted into a 96-well plates. Growth (OD_612__nm_) at 41.5°C (±0.3°C) was monitored every 20 min for at least 3 days, using a TECAN Infinite F500 reader. Optical density values were corrected with the average background optical density measurement of abiotic medium. Biological replicates were performed in triplicate.

Growth analyses of *S. acidocaldarius* were performed manually as follows: exponentially growing cells (Brock medium supplemented with uracil) (Brock et al., 1972; Wagner et al., 2012) were diluted in pre-warmed Brock medium containing various relative amounts of 4TU/uracil (final concentration 180μM) and incubated at 65°C with agitation. Optical density (OD_600__nm_) was measured at regular time interval.

### Labeling with 4TU

For *H. volcanii*, WT cells H26 (uracil auxotroph – Δ*pyrE2*) and “pop-in” mutants (uracil prototroph – *pyrE2*) were first grown in Hv-Ca^+^ medium in presence or absence of uracil (400 μM), respectively (H26: +URA [400 μM], “pop-in”: -ura).

For 4TU pulse labeling experiments, exponentially growing cells were then transferred to fresh Hv-Ca^+^ media containing the indicated amount of 4TU [typically 75% 4TU (300 μM) and 25% uracil (100 μM)].

For pulse-chase experiments, cells were first grown in presence of 4TU and transferred to media Hv-Ca^+^ supplemented with uracil and lacking 4TU for the indicated time.

For *S. acidocaldarius*, MW001 cells were grown in Brock media supplemented with uracil (180 μM) (Brock et al., 1972; Wagner et al., 2012). For pulse labeling experiments, exponentially growing cells were transferred to pre-warmed Brock media containing 75% 4TU (135 μM) and 25% uracil (45 μM).

For each sample time point, 2 ODs (OD_600__nm_) were collected from cultures ranging between 0.4 and 0.8 OD (OD_600__nm_), centrifuged and frozen or immediately processed further.

### Total RNA Extraction

Total RNA was extracted using the hot-phenol extraction procedure as previously described (Schmitt et al., 1990).

### RNA Biotinylation

For RNA biotinylation, typically 20–100 μg total RNA was labeled in the dark in presence of 50 μg HPDP-biotin (Pierce) or 5 μg MTSEA-biotin-XX (Biotium – 90066) in 10 mM Tris-HCl pH 7.4; 1 mM EDTA pH 8 for 2 h (HPDP-biotin) or 30 min (MTSEA-biotin-XX) (Duffy et al., 2015). Biotinylated RNAs were purified by the hot-phenol extraction procedure (Schmitt et al., 1990).

### Slot Blot and Northern Blot Analysis

Total RNA (typically 5–10 μg) was either separated by denaturing agarose gel electrophoresis in the absence of ethidium bromide or Syber-safe and transferred onto a positively charged nylon membrane (Ferreira-Cerca et al., 2005) or directly immobilized on nylon membrane using a vacuum slot-blotter (Millipore). RNAs were UV cross-linked twice with 0.5 J/cm2.

### Detection of RNA

For radioactive detection of bulk steady-state rRNA the following radiolabeled probes oHv194 5′-^32^P-CCTCGGCTGATTGACAGTGCC-3′, and Saci_022 5′-^32^P-CCTTTCGGTCGCCCCTACTC-3′, antisense of the mature *H. volcanii* and *S. acidocaldarius* 23S rRNA, respectively, were used.^32^P labeling of oligo probes, blot hybridization, and radioactive signal acquisition were performed as previously described (Ferreira-Cerca et al., 2005).

For fluorescent detection of 4TU labeled RNA, membranes were blocked for 20 min in blocking solution (1X PBS pH 7.5, 1 mM EDTA pH 8) containing 10% (w/v) SDS under mild agitation. Subsequently, the membranes were incubated at room temperature with IR-dye conjugated Streptavidin (1:10,000 dilution in blocking solution containing 10% SDS – IRDye 800CW Streptavidin, Pierce) for 20 min in the dark under mild agitation. Membranes were then washed with blocking solution containing decreasing amounts of SDS, twice each with 10, 1, and 0.1% SDS, for 10 min each. Labeled RNAs were visualized using a LI-COR Odyssey imaging platform.

### Relative Quantification

Fluorescence and radioactive signals were quantified with ImageJ. 4TU-labeled 23S rRNA signal intensity (fluorescence signal) were normalized to the bulk 23S rRNA signal (radioactive signal).

### Affinity Purification of Bio-Tagged-RNA

Exponentially growing wild type *H. volcanii* cells (H26) were diluted into pre-warmed enhanced casamino acids supplemented either with uracil and 1 mM tryptophan or supplemented with 4TU/uracil with or without 1 mM tryptophan. After 45 min of incubation 4 OD_600__nm_ of cells were harvested. Total RNA was extracted as described above. Residual genomic DNA was digested in presence of RQ1 DNAse (Promega) and RNasin (NEB) as recommended by the manufacturer. The RNA was purified by hot-phenol extraction and biotinylated as described above. Purified RNA (25 μg) was resuspended in buffer B (20 mM Tris–HCl pH 7.6, 150 mM NaCl, 5 mM MgCl2, 1 mM DTT) (Nielsen et al., 2011) and incubated for 2 h on a rotating wheel at 4°C with high-capacity Streptavidin agarose beads (Pierce) in a total volume of 800 μl buffer B. Immobilized RNA was washed three times for 10 min on a rotating wheel at 4°C with 800 μl buffer B. Competitive RNA elution was performed twice in batches using 500 μl buffer B supplemented with 2.5 mM D-desthiobiotin (IBA) at 4 and 23°C, respectively. RNA was purified using hot-phenol extraction as described above.

### Quantitative RT-PCR Analysis

Reverse transcription (SuperScript III – Invitrogen) was performed according to the manufacturer’s instructions in presence of 0.2 μg random primers (Agilent). Complementary DNA synthesis was performed with a pre-incubation at 25°C followed by 60 min at 42°C.

Quantitative PCR was performed with previously published primers, complementary to the tryptophanase ORF Trpase_R2 5′-ACACCGGTTCGAGCCGCGACG-3′, Trpase_F2_RT 5′-TTCGCGTTCCCCGGCACCGAC-3′ (202 bp) and the ribosomal protein L10 ORF RibL10-H.v. 5′-CCGGTCGCCTGCTTGTTCTCGCG-3′, RibL10-H. v. 5′-CCGAGGACTACCCCGTCCAGATTAGCCTG- 3′ (187 bp) (Large et al., 2007).

Each PCR reaction was performed in a 20 μl reaction containing 2 μl 10X PCR-Buffer (Qiagen), 0.8 μl 25 mM MgCl2, 0.16 μl 25 mM dNTP-mix, 0.08 μl (5 U/μl) HotStar Taq (Qiagen), 0.25 μl SYBR green I dye stock (Roche, diluted in DMSO 1:400000), 4 pmol of each primer and 4 μl of template cDNA.

PCR reactions and SYBR green I dye fluorescence acquisition were performed with a Rotor-Gene 3000 system (Corbett Research/Qiagen). Relative quantification analysis was performed using the comparative analysis software module (Rotor-gene 6 – Corbett Research/Qiagen). Relative levels were calculated according to the 2^-ΔΔC^_T_ method (Livak and Schmittgen, 2001) and using the ribosomal protein L10 mRNA level as reference (Large et al., 2007). Serial dilutions of the samples were run in triplicate to ensure accuracy of the data.

## Author Contributions

RK, CK, and SF-C designed and performed all the experiments and interpreted the results. SF-C supervised the study and wrote the paper. All authors critically commented on the manuscript.

## Conflict of Interest Statement

The authors declare that the research was conducted in the absence of any commercial or financial relationships that could be construed as a potential conflict of interest.

## Footnotes

1 http://archaea.ucsc.edu/cgi-bin/hgGateway?db=haloVolc1

## Supplementary Material

FIGURE S1 **Detection of 4TU with HPDP-biotin and MTSEA-biotin-XX.** *H. volcanii* (H26) cells were grown for several generations either in medium containing a mixture of 4-thiouracil and uracil (4TU/U – 3:1) or in medium solely containing uracil (U). Nucleic acids were analyzed as described in the Section “Materials and Methods.”

FIGURE S2 **Generation of uracil prototroph strain.** The upstream and downstream region of the HVO_0569 open reading frame were amplified by PCR and cloned into the integrative vector pTA131 and transformed in H26 cells. Recombination events leading to genomic integration of the *pyrE2* gene were selected on Hv-Ca^+^ plates lacking uracil.
