# Supplementary figures and images for: Toward Time-Resolved Analysis of RNA Metabolism in Archaea Using 4-Thiouracil
Source: Front Microbiol. 2017 Feb 24;8:286. doi: 10.3389/fmicb.2017.00286 (PMC5323407; doi:10.3389/fmicb.2017.00286)

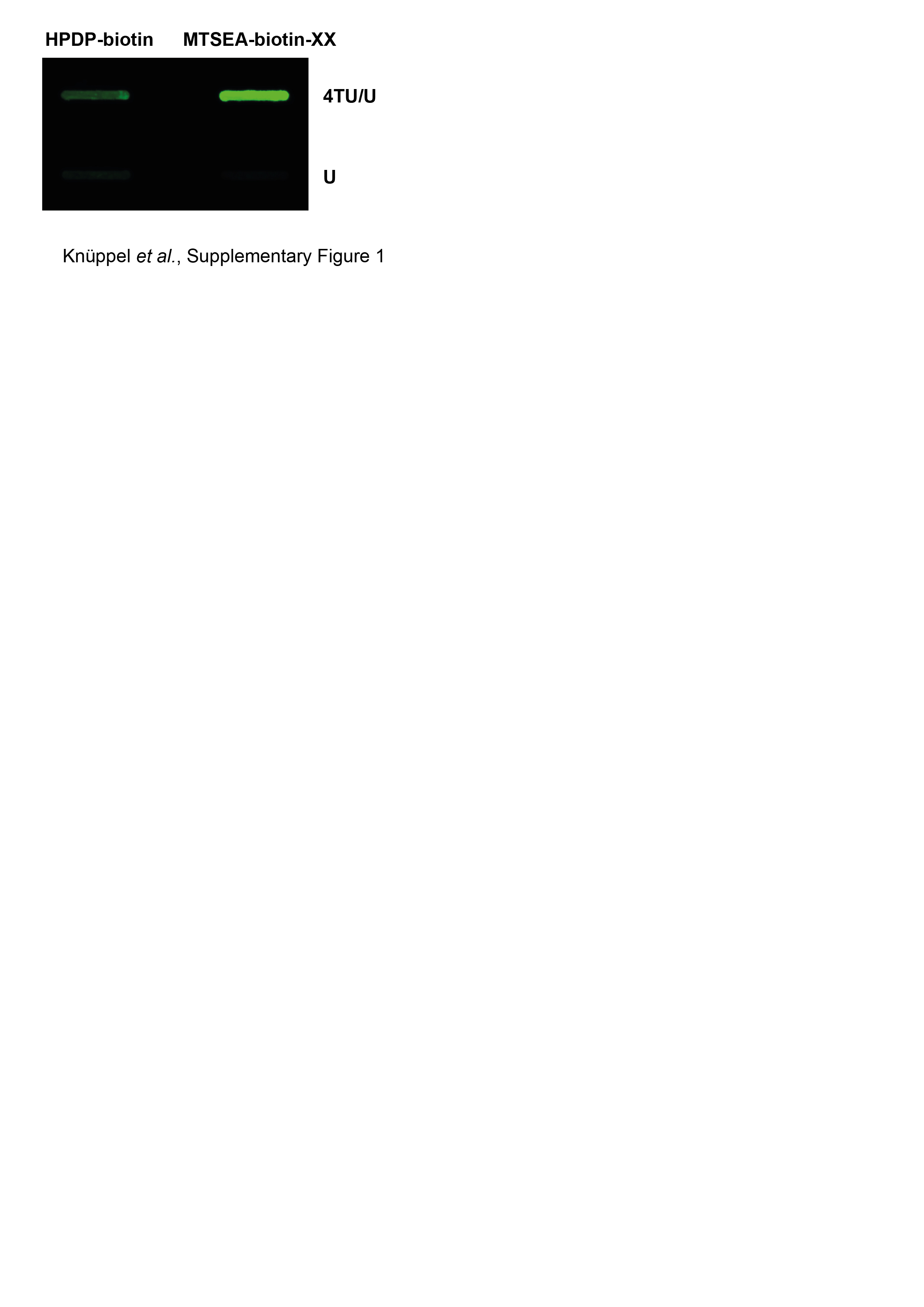

Supplement: FIGURE S1 — Detection of 4TU with HPDP-biotin and MTSEA-biotin-XX. H. volcanii (H26) cells were grown for several generations either in medium containing a mixture of 4-thiouracil and uracil (4TU/U – 3:1) or in medium solely containing uracil (U). Nucleic acids were analyzed as described in the Section “Materials and Methods.” [file Image_1.JPEG]

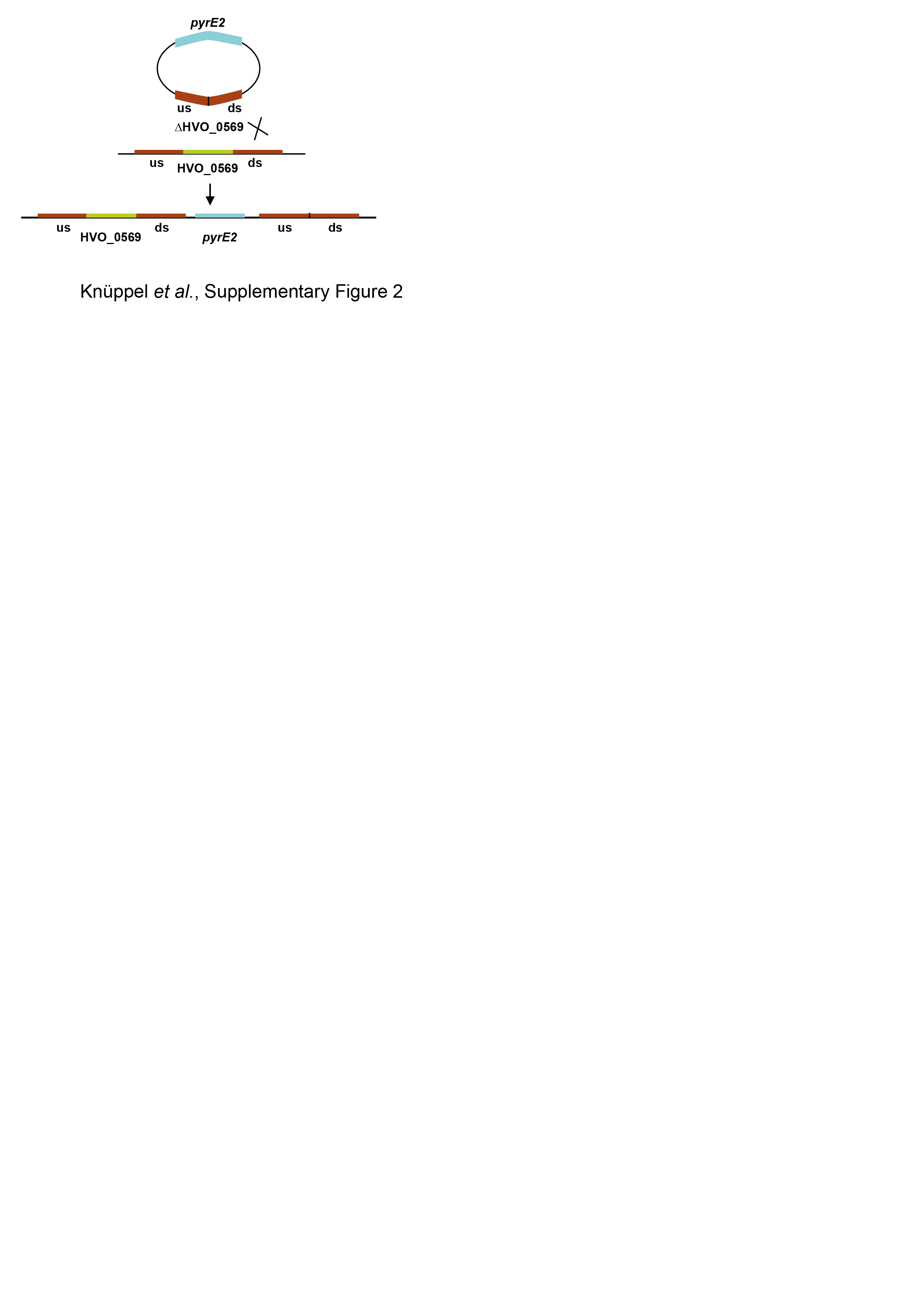

Supplement: FIGURE S2 — Generation of uracil prototroph strain. The upstream and downstream region of the HVO_0569 open reading frame were amplified by PCR and cloned into the integrative vector pTA131 and transformed in H26 cells. Recombination events leading to genomic integration of the pyrE2 gene were selected on Hv-Ca+ plates lacking uracil. [file Image_2.JPEG]
